# Employing Community Voices: Informing Practice and Programming through Camden Healthy Start Focus Groups

**DOI:** 10.1007/s10995-017-2382-0

**Published:** 2017-11-11

**Authors:** Dianne R. Browne, Sherolde Hackett, Allison Burger

**Affiliations:** 1 SNJPC Camden Healthy Start, 808 Market Street, Camden, NJ 08102 USA; 2 Southern New Jersey Perinatal Cooperative, 2500 McClellan Avenue, Suite 250, Pennsauken, NJ 08109 USA

**Keywords:** Healthy Start, Access to care, Barriers to care, Women’s health, Client attitudes

## Abstract

*Objectives* Women living in communities with low-socioeconomic status, substandard healthcare, and ongoing exposure to social disparities encounter barriers to healthcare, often making it difficult to access health services. Barriers may stem from provider interactions with clients, conditions of the healthcare facility, or even language barriers. This prompts a call for providers to be keenly aware of the obstacles women encounter when attempting to access services. *Methods* In an effort to facilitate better access to services, Camden Healthy Start conducted six focus groups. Thirty-nine women between the ages of 22–56 participated. A total of 39 questions were posed to participants about health behavior, health services, pregnancy, reproductive health, and barriers to accessing services. Each 2 h session was audio recorded, translated and transcribed. Following the format of the *Women’s Health: Attitudes and Practices in North Carolina Focus Group Research*, responses were analyzed and themes emerged.* Results* This article discusses characteristics of healthcare services and cultural insensitivity that impact women’s access and act as barriers to care. The results signal the need for Healthy Start to apply a more relational engagement when providing services.* Consideration for Practice* Relational engagement includes getting to know the client as a person first, respecting their rights to autonomy in the decision making process, and demonstrating an understanding of the client’s culture and inclusion of their voices in the conversation.

## Significance

Celebrating 25 years, Healthy Start has a history of establishing relationships with poor and underserved women that facilitate successful navigation of barriers in health services. This article draws attention to the centrality of developing relationships with clients in order to reduce client barriers to accessing services. It also serves as a call for new programs to get to know the communities they serve.

## Introduction

Good health is a right of all Americans. The World Health Organization asserts all humans have a fundamental right to the highest attainable standard of health regardless of race, ethnicity, and socioeconomic standing (World Health Organization 2016). However, minority families living in American communities with low-socioeconomic status and ongoing exposure to social disparities experience less than impressive positive health outcomes. Camden Healthy Start (CHS) serves women in such a community. Poor birth outcomes such as low birth weight, premature birth, and maternal morbidity are common among families residing in Camden, New Jersey.

Communities funded under the Healthy Start initiative are disproportionately impacted by poor health (HRSA 2016) and high infant mortality rates. The New Jersey Department of Health (2016) reports that the infant mortality rate in the city of Camden is more than twice the statewide rate. As seen in Table 1, the rate among African American and Hispanic women in Camden is more than three times that of white women in the state. These numbers are of concern to CHS as African American and Hispanic women make decisions not to seek prenatal care or follow through with postpartum check-ups. It was critical to understand the barriers preventing women from accessing and receiving services so CHS could avoid pitfalls and foster women’s participation in its services.

**Table 1:** Comparison of infant mortality rates in New Jersey

Race/ethnicity	City	State
White	^a^	2.9
Black	14.3	10.3
Hispanic	9.8	4.3
Total	11.8	4.7

Infant mortality rates in the city of Camden and the state of New Jersey from 2010 to 2012

^a^Rate cannot be calculated with reliability or precision due to low numbers

## Purpose

Healthy Start programs have historically worked with women in various stages of reproductive development (HRSA 2016). Longtime engagement with women has resulted in a historical understanding of inventive and creative ways women successfully overcome barriers to receiving good healthcare. However, less seasoned Healthy Start programs may not have such experience guiding their practice. Similarly, healthcare providers serving Healthy Start clients may not understand that an appreciation of client barriers, such as transportation, customer service, and provider manners, are ways of demonstrating receptiveness to the issues and concerns of women. In order to better understand the barriers that prevent women from accessing health services in Camden, CHS held a series of focus groups with women. The purpose of the focus groups was to solicit the perspectives of women about perceived barriers to obtaining care for their babies, children, and themselves in an effort to avoid perpetuating barriers to health services in the CHS project.

## Methods

Authors began the study by brainstorming attitudes and beliefs about barriers to women accessing health services based on their professional experience of more than 25 years in the field. Barriers discussed included: transportation, family pressures, cultural remedies vs professional services, difficulty getting appointments, and homelessness.

Next, authors reviewed reports of other Healthy Start programs that studied women’s health services. Two reports, the Oklahoma Preconception and Pregnancy Health Focus Groups Summary Report and Recommendations (Oklahoma State Department of Health 2011) and the North Carolina State Infant Mortality Collaborative (NCSIMC) Report on Women’s Health (Burnet 2005), were particularly informative about using focus groups to assess women’s attitudes and beliefs about healthcare.

Women in Camden were recruited for the focus groups by community based agencies. Flyers with the theme *Camden Lives Matter* were developed by CHS and distributed by the recruiting agencies. Along with details regarding location and time, the flyer included a focus group description and notice that a meal and incentive would be provided to those who complete the session. Table 2 provides racial and ethnic demographics of the 39 participants. The target population was women of child bearing age, however participants ranged in age from 22 to 56.

**Table 2:** Racial and ethnic demographics of healthy start focus group participants

n = 39	
Race/ethnicity	Number
Hispanic	22
Black/African American	10
Caucasian	1
Non-Hispanic Multiracial	1
Hispanic Multiracial	1
No response	4

Written consent forms in English or Spanish were provided to attendees, based on their language of choice. Consent forms were read aloud to attendees and questions were elicited and answered. All participants gave their signed consent prior to participating in the focus groups. Once the participant group was established, ground rules were created and definitions of terms provided. Six focus groups were held, with four to ten women in each session. Four of the groups were conducted in English and two in Spanish. Table 3 provides additional characteristics of the participants.

**Table 3:** Demographic characteristics of focus group participants

n = 39					
Community partners	# Of participants	Parental status	Pregnant women	Household income < $15,000	OB/GYN check in last 12 months
Promise Neighborhood Family Success Center	10	5 Non-single women 5 Single women	1	5	6
Camden County Women’s Center	5	2 Non-single women 3 Single women	0	3	4
Hispanic Family Center	6	4 Non-single women 2 Single women	1	5	4
St. Josephs Childcare Center	8	7 Non-single women 1 Single women	1	6	8
Hispanic Family Center	6	4 Non-single women 2 Single women	1	5	4
First Baptist Church	4	1 Non-single woman 1 Single woman 2 Non-parents	0	1	4

### Focus Groups

Facilitators conducted semi-structured interviews using an interview guide from the NCSIMC. Sessions were recorded with digital recorders and co-facilitators took detailed notes. The discussions surveyed participants’ understanding of health behavior, health services, pregnancy, reproductive health, and barriers to accessing services, using 39 total questions. At the close of the session, participants completed a health and demographic questionnaire and received a $25 gift card.

Each recording was translated and transcribed. A case-oriented approach was used to analyze the data. This approach attempts to understand a phenomenon which, in this case, was participants’ real or perceived barriers to health services (Schutt 2012). The analysis focused on identifying all key concepts mentioned by respondents under each topic and subtopic and identifying the most illustrative quotes. The manuscript is not based upon clinical study or patient data.

## Results

The analysis of participant responses identified key themes for each topic and highlighted barriers to health and health services that were somewhat different from what CHS staff had postulated. As anticipated, women acknowledged adopting preventive health behaviors and referenced a struggle in meeting basic needs that interfered with taking care of their health. However, with the exception of transportation, the needs that participants identified as unmet departed from what CHS staff expected. Participants identified waiting for Medicaid, fear of deportation, and not feeling well enough to go for care due to pain. Whereas unmet needs are challenging for programs to overcome, some of the barriers presented by participants were ones that service providers, such as CHS, have direct control over. These included poor customer service, discomfort with where services were provided, racial/ethnic disparity, and a lack of respect.

### Customer Service

Poor customer service was referenced in several focus groups, varying by location and issue. Participants wanted to be informed of appropriate services and follow up related to their medical appointments. They noted the “lack of friendly, knowledgeable staff members” at some facilities. When participants were asked how staff could improve services, participants’ suggestions centered on maintaining a positive attitude, “Talk to me the way you want me to talk to you.” Another participant suggested that providers need to ask “how are you” and learn how to treat the whole woman. Other participants mentioned long wait times for appointments: “…you’ve got an appointment for 10:00 and they’ve got 10 people for an appointment at 10:00.” Long wait times are disappointments to clients’ expectation of services, “Like when they tell you, you need to be there at 9 and they don’t even see you until 11:30.”

### Conditions at Facilities

Women described the unacceptable conditions of healthcare facilities, “If they trashy or you see roaches running around I’m not coming back to nobody’s doctor office.” Another participant discussed changing providers because of facility conditions, “…I hated going there…just looked dirty, the walls were bare…As soon as I turned 16 I was asking my friends, who’s your primary care doctor?” Another commented on the atmosphere of the facility, “they were so ghetto that I don’t go to there… people just sitting there… with their shoes off, all loud and coughing and the security was all hands off.”

### Racial and Ethnic Disparity

Participants shared stories about racial and ethnic discrimination and lack of accommodation for Spanish-speaking clients. One client referenced her ethnicity and immigrant status as barriers, “I don’t know if it’s from being Hispanic or an immigrant here, sometimes they don’t treat you the same.” Another shared an experience she had while waiting with her brother to receive services, “And she pointed at us and said to someone else… since they’re illegals, [they] have to wait longer.” Women talked about others in the waiting rooms being frustrated because of language barriers, “I’ve seen women crying because nobody understands them.”

Providers’ lack of cultural competence and sensitivity to language differences offered a different kind of barrier. One woman said providers’ explanations were not clear. “I ask and I be like, can you break that down because I don’t even know what that is?” Another shared language concerns, “It sounds like they’re talking [another language] to me. I’m like, what, what? They use some big words, you know… I gotta write that down.”

### Respect

Some of the stories described barriers related to being disrespected. Women rejected “being talked at,” by the provider or the provider “not looking at you [the patient] but…in their iPad.” Participants talked about the lack of support in the relationship, “[healthcare providers] ask you… they don’t really encourage you… to take care of [your condition] every 6 months.” One attendee talked about how respect translated to service, “if you don’t treat me with the utmost respect, you’re not going to be acting to take care of my business the way that you should either.” Responses revealed women’s discontent, fears and concerns that were tantamount to barriers to their accessing or returning for services.

## Discussion

CHS sought to learn how to be proactive in preventing barriers to women’s access to services. Responses from focus group participants departed from CHS staff expectations, prompting CHS to expand its literature review to better discern the meaning behind women’s comments. In particular, participants noted a greater number of ways that racial and ethnic stereotyping and discrimination can be barriers to services. Lori et al. (2011) described African American women’s desire for specific provider characteristics suggesting that determinants of health disparities tend to be social or relative to the situation in which service is provided. Therefore, CHS’s reaction to clients’ attributes, such as the woman’s age, marital status, race/ethnicity, communication ability, or fear was considered more closely.

Hispanic women less acculturated to Western medical practices face more disparities in the provision of services than do native non-immigrant Hispanic women. Some of this relates to a belief that staff is in charge or in control (Roncancio et al. 2011). Fear of deportation, loss of their children, having limited support systems, and experiencing language barriers (Pérez-Escamilla et al. 2010) limit women’s access to services. Understanding the client’s story, explaining aspects of services and assuring her of a procedure’s safety to her and her children may reduce barriers.

Benkert et al. (2006) found that experiencing racism directly influences patient satisfaction, and argued that it is imperative for a patient to have a choice when selecting their primary care provider. When a client selects her own provider, her increased satisfaction strengthens her resolve to adhere to physician recommendations for follow-up care. CHS considered this sound advice when matching participant and worker.

### Customer Service

When examining its own customer service, CHS considered how clients were recruited and engaged in services. Since CHS is a home visiting model, prioritizing clients’ comfort by meeting them in their home, in the community or in the CHS office was important. CHS responded to the importance of staff reflecting the community by hiring individuals that look like the residents, speak the same languages as clients, and when possible, live in the city. CHS also prepares bilingual staff to be attentive to differences in language translation, and affirm the importance of a client’s culture and customs in the decision making process.

### Client-Focused Services

CHS was interested in hearing unfiltered and direct client voices to improve client-focused services. Client-focused services are strengths-based approaches designed to be intentional about meeting a client’s need, involving her in the decision-making process, and considering her frame of reference be it race, culture or language in the process of care (Epstein and Street 2011). Client-focused services support the development of personal relationships between staff and client (Campinha-Bacote 2011). CHS was intentional about getting to know the client as a person first, respecting clients’ rights to decision making autonomy, demonstrating an understanding of the client’s culture and inclusion of their voices in the conversation (Kaminsky 2013). CHS’ client-focused approach included asking participants what they wanted from the program; providing flexible meeting options for participants to meet with workers; and providing weekend events that included the entire family.

### Mindfulness

Mindfulness is the ability to consider a situation from the client’s experience without judging or stereotyping (Escuriex and Labbe 2011; Langer and Moldoveanu 1999). When visiting a woman’s home, when listening to her story, when meeting her in the community, mindfulness is present in the greeting, invitation and delivery of any services or materials.

Staff is mindful of the countries from which women come, being sure to use familiar language and terms. As one participant put it “…it’s important that healthcare professionals are involved in the community they serve… they kind of understand the lifestyles of the individuals in the communities they serve.”

Staff is mindful of life outside the program. Each visit begins by having the client share what has happened since their last meeting and addressing any immediate needs. Staff close each visit by restating next steps and seeking input from the client. Staff use positive interpersonal skills, including making eye contact and giving the client their full attention, in every encounter. CHS strives to engage with clients in the manner they desire by demonstrating an understanding of the client’s culture and including clients’ voices in the provision of services.

## Conclusion

Not all access barriers and provider interactions are the same, there were stories of positive exchanges between clients and providers. Nevertheless, a key lesson learned was the significance of the relationship between client and staff. Future research examining skills and techniques to build relationships and improve communication across racial and ethnic groups in client care is needed.

Limitations of the study include results that are not generalizable to all Healthy Start programs as program designs and models differ across the country. CHS did not recruit the women in the study, thus women outside of the childbearing age were included. The focus group format limits the applications of this research, but provides a basis to review current practices. Further studies by Healthy Start projects could more specifically investigate racial/ethnic disparities in women’s health services.

Healthy Start needs to be keenly aware of all obstacles women encounter when accessing care. Awareness and sensitivity to those being treated and genuine interest in client needs strengthens client-focused care. Based on the results of the focus groups, CHS reviewed its practices to ensure that they aligned with the project’s growing understanding of effective ways to reduce barriers to participation.

While veteran Healthy Start programs are experienced in developing relationships and helping women overcome barriers, newer programs without a historical advantage would benefit from identifying, comprehending and analyzing barriers from the client’s point of view. Taking the time to do this prior to program delivery supports the creation of client-focused services and continue to strengthen supportive services.
